# Diet analysis using generalized linear models derived from foraging processes using R package *mvtweedie*

**DOI:** 10.1002/ecy.3637

**Published:** 2022-03-16

**Authors:** James T. Thorson, Mayumi L. Arimitsu, Taal Levi, Gretchen H. Roffler

**Affiliations:** ^1^ AFSC, NMFS, NOAA Seattle Washington USA; ^2^ U.S. Geological Survey Alaska Science Center Juneau Alaska USA; ^3^ Department of Forest Ecosystems and Society Oregon State University Corvallis Oregon USA; ^4^ Alaska Department of Fish and Game Douglas Alaska USA

**Keywords:** diet, foraging theory, generalized additive model, generalized linear model, metabarcoding, point process, Poisson process, predation, Tweedie distribution

## Abstract

Diet analysis integrates a wide variety of visual, chemical, and biological identification of prey. Samples are often treated as compositional data, where each prey is analyzed as a continuous percentage of the total. However, analyzing compositional data results in analytical challenges, for example, highly parameterized models or prior transformation of data. Here, we present a novel approximation involving a Tweedie generalized linear model (GLM). We first review how this approximation emerges from considering predator foraging as a thinned and marked point process (with marks representing prey species and individual prey size). This derivation can motivate future theoretical and applied developments. We then provide a practical tutorial for the Tweedie GLM using new package *mvtweedie* that extends capabilities of widely used packages in R (*mgcv* and *ggplot2*) by transforming output to calculate prey compositions. We demonstrate this approach and software using two examples. Tufted Puffins (*Fratercula cirrhata*) provisioning their chicks on a colony in the northern Gulf of Alaska show decadal prey switching among sand lance and prowfish (1980–2000) and then Pacific herring and capelin (2000–2020), while wolves (*Canis lupus ligoni*) in southeast Alaska forage on mountain goats and marmots in northern uplands and marine mammals in seaward island coastlines.

## INTRODUCTION

Ecologists often collect data regarding the proportion of a total belonging to different categories (called “compositional data”). The multivariate structure of compositional data is informative regarding trophic relationships of consumers and producers, competition dynamics, and the flow of energy in food webs (Bonin et al., 2020; Shi et al., 2021). Many methods are used to measure the diet proportions of consumers including morphological identification of prey in stomach contents or feces, ratios of stable isotopes or fatty acids, and molecular approaches including DNA metabarcoding (Roffler et al., 2021; Shi et al., 2021). Potential applications of compositional diet data range from defining the foraging niche of individuals, populations, or species to ecosystem‐based management informed by trophic models.

Compositional data are often analyzed using models that are difficult to parameterize and interpret. For example, a Dirichlet distribution (the multivariate generalization of the beta distribution for proportions) can be fitted to proportions (ranging continuously from 0 to 1) derived from biomass samples within stomach contents (Ainsworth et al., 2010; Maier, 2021). However, this approach has several limitations, including:

1. The Dirichlet distribution does not support a value of 0 or 1, so the distribution cannot account for instances when the prey is either absent or the only species within a given sample. To rectify this, prey responses must be changed from 0/1 to for example, 0.0001/0.9999 (or some other arbitrary bounds) prior to analysis.
2. It is not clear how to incorporate covariates in an ecologically interpretable manner within the Dirichlet distribution. Analysts have incorporated covariates in a log‐linked linear predictor for the concentration parameter for each prey (Maier, 2021). However, there is no explicit link between this concentration parameter and variables that are interpreted in foraging theory, for example, prey densities, predator energetic condition, or functional‐response parameters.

In response to the first limitation, Moriarty et al. (2016) developed an alternative method that separately models the probabilities that 0% or 100% of a given stomach belong to each species (also see Douma & Weedon, 2019). This mixture model eliminates the need to pre‐specify a lower/upper bound on encounters for any individual prey species. However, this solution in turn introduces many additional parameters, representing the probability that a given prey is 0% or 100% of stomach contents, in addition to parameters governing the expected density for intermediate values.

For these reasons, we instead seek to derive a model for compositional analysis that uses raw sampling data (without transformation prior to analysis), while also being parsimonious and derived directly from variables used in foraging theory. In particular, we build upon efforts to recast ecological models as thinned and marked point processes (Illian et al., 2008). We also seek to provide a simple code example for two contrasting cases using widely used software that is familiar to ecologists. We therefore develop a new R package *mvtweedie*, which provides a new *predict* S3 class that transforms output from widely used R packages (*mgcv* for GAMs or *glmmTMB* for GLMMs) to predict prey compositions for any new set of covariates. These covariates can represent, for example, predator energetic condition, spatiotemporal variation in habitat predicting prey densities, as well as correlated residual variation. We then show how to explore this output using high‐level graphical tools (*ggplot2*).

## METHODS

### Thinned and marked Poisson processes

We first briefly introduce the theory of a thinned marked point process (Illian et al., 2008). These processes provide a useful mathematical representation for animal foraging, but have received surprisingly little attention from ecologists for this or other uses (Illian & Burslem, 2017). Modifying notation from Illian et al. (2008), a point process has an intensity function $\Lambda \left(s\right)$ for every location s with units of numbers per area, and the number of individuals within a subset A of the spatial domain is a random variable $N\left(A\right)$ with expectation drawn from the intensity function, $E\left(N\left(A\right)\right)=\lambda \equiv \int_{A}\Lambda \left(s\right)ds$.

Our presentation combines the following four elaborations (see Appendix S1 for details) of this basic point process to make it relevant for estimating predator feeding habits:

1. Prey encounters are distributed randomly at local scales (a Poisson process);
2. Prey densities are governed by spatial processes and environmental covariates that can be expressed as a log‐linked linear model (a log‐Gaussian‐Cox process);
3. Prey animals have “marks” representing species and size (a marked point process);
4. Predators attack and consume a subset of local prey encounters (a thinned point process).

This thinned and marked Poisson process includes several well‐known cases as submodels. In particular, we note the Multinomial‐Poisson transformation as a widely used submodel (Appendix S2) before introducing the Tweedie generalized linear model (GLM) that is our primary focus.

### Tweedie distribution for thinned and marked Poisson process

The thinned and double‐marked Poisson process results in a convenient approximation for biomass responses when individual prey size is assumed to follow a Gamma distribution. Given that we have multiple prey categories, we have a Poisson process for counts $N_{\mathrm{ic}}$ of each prey type c in each sample i of predator food habits, $N_{\mathrm{ic}}∼\text{Poisson}\left(\lambda_{\mathrm{ic}}\right)$, and a gamma distribution for the mass $B_{\mathrm{icn}}$ of the *n*th of these $N_{\mathrm{ic}}$ animals, $B_{\mathrm{icn}}∼\text{Gamma}\left(k_{c},\theta_{c}\right)$, using the shape‐scale parameterization where $k_{c}=v_{c}^{-2}$ and $\theta_{c}=w_{c}v_{c}^{2}$ given that $w_{c}$ is the average biomass and $v_{c}$ is the coefficient of variation in individual biomass for each prey c.

Total biomass $Y_{\mathrm{ic}}$ for each prey c in sample i is then the sum of individual biomass $B_{\mathrm{icn}}$ for each of these $N_{\mathrm{ic}}$ animals of prey c in that sample. This is called a compound Poisson‐gamma distribution for $Y_{\mathrm{ic}}=\sum_{n=1}^{N_{\mathrm{ic}}}B_{\mathrm{icn}}$. Usefully, this compound Poisson‐gamma process can be exactly expressed as a Tweedie distribution (Foster & Bravington, 2013),

(1) $$Y_{\mathrm{ic}}∼\text{Tweedie}\left(\mu_{\mathrm{ic}},\phi_{\mathrm{ic}},p_{c}\right)$$

where the Tweedie mean parameter $\mu_{\mathrm{ic}}=\lambda_{\mathrm{ic}}w_{c}=E\left(Y_{\mathrm{ic}}\right)$ represents the expected thinned biomass for each prey, that is, the product of thinned local abundance $\lambda_{\mathrm{ic}}$ and average weight $w_{c}$ for prey c (see Appendix S3: Figure S1). We call $\phi_{\mathrm{ic}}$ the scale parameter and $p_{c}$ the power parameter, noting that $\mathrm{Var}\left(Y_{\mathrm{ic}}\right)={\phi_{\mathrm{ic}}\mu }_{\mathrm{ic}}^{p_{c}}$ (Foster & Bravington, 2013). These three Tweedie parameters can be calculated from thinned local prey abundance and size $\mu_{\mathrm{ic}}=\lambda_{\mathrm{ic}}k_{c}\theta_{c}$, $\phi_{\mathrm{ic}}=\theta_{c}\left(k_{c}+1\right)\mu_{\mathrm{ic}}^{\frac{-1}{k_{c}+1}}$, and $p_{c}=\frac{k_{c}+2}{k_{c}+1}$, and all three vary among samples and/or prey.

### Estimation using a Tweedie GLM

We next show how to fit this Tweedie distribution (representing a thinned and marked Poisson process) as a GLM. This involves specifying that power and scale parameters are constant across all categories and samples ($\phi_{\mathrm{ic}}=\phi$ and $p_{c}=p$). The resulting model can then be conveniently fitted to data as

(2) $$\mathrm{vec}\left(Y_{\mathrm{ic}}\right)∼\text{Tweedie}\left(\mathrm{vec}\left(\mu_{\mathrm{ic}}\right),\phi ,p\right)$$

where operator $\mathrm{vec}\left(.\right)$ vectorizes a matrix such that for example, $\mathrm{vec}\left(Y_{\mathrm{ic}}\right)$ is the vector formed by stacking columns of matrix Y, and the same ordering is used for $\mathrm{vec}\left(\mu_{\mathrm{ic}}\right)$. This can be fitted using standard software for a Tweedie GLM with prey as a category, for example, using R packages *mgcv* (Wood, 2006) or *glmmTMB* (Brooks et al., 2017).

Estimation using a Tweedie GLM allows us to rapidly explore covariates predicting thinned biomass density for each prey:

(3) $$\log\left(\mu_{\mathrm{ic}}\right)=\alpha_{i}+\sum_{k=1}^{n_{k}}\beta_{\mathrm{kc}}x_{\mathrm{ik}}$$

where $\alpha_{i}$ represents the product of area‐swept and thinning rate that underlies sample i (see Appendices S1 and S2 for more details). In some designs, it is reasonable to assume that all samples arise from the same area swept and have equivalent thinning rates, such that $\;\alpha_{i}=\alpha$. In other cases, $\alpha_{i}$ could itself be modeled as a function of predator body size or other covariates, to again control for unknown differences in area swept and thinning rate associated with each sampled predator.

The Tweedie GLM then allows proportions to be back‐calculated as

(4) $$\pi_{\mathrm{ic}}=\frac{\exp\left(\sum_{k=1}^{n_{k}}\beta_{\mathrm{kc}}x_{\mathrm{ik}}\right)}{\sum_{c^{*}=1}^{n_{c}}\exp\left(\sum_{k=1}^{n_{k}}\beta_{kc^{*}}\;x_{\mathrm{ik}}\right)}$$

This shows that proportions $\pi_{i}$ in the thinned and marked Poisson process can be fitted to raw samples (e.g., prey biomass in stomach contents) using a standard GLM framework, as an alternative to converting samples to proportions prior to analysis. Covariance in $Y_{i}$ arising from satiation or multi‐species functional responses could be approximated via random effects for $\pi_{i}$ relative to prey densities at each location, and we leave this as a topic for future exploration. We provide an alternative interpretation for the joint distribution of samples $Y_{i}$ for all prey c as arising from a “multivariate Tweedie” distribution in Appendix S3.

### Simulation example

We visualize this thinned and double‐marked Poisson process using a simplified simulation experiment involving three prey and three foraging habitats, where each foraging habitat has distinct patch size A. We use this experiment in part to visualize the magnitude of error that may arise when approximating a thinned and double‐marked Poisson process with a Tweedie distribution.

To do so, we first simulate locations for 1000 individuals of each prey from density function $\Lambda_{c}\left(s\right)$, where $\Lambda_{c}\left(s\right)$ is specified as a bivariate normal distribution centered at different locations for each prey, and each prey has a gamma distribution for individual mass parameterized with mean $w_{c}=1.2\;\mathrm{kg}$ and coefficient of variation $v_{c}=1$. We visualize the spatial distribution and associated body size for each prey in a single realization of this experiment, and record the biomass for each prey within each of the three foraging areas.

We then repeat this simulation 1000 times to generate the expected distribution for prey biomass within each foraging area. We calculate the Tweedie parameters ($\mu_{c}\left(s\right)$, $\phi_{c}\left(s\right)$ and $p_{c}\left(s\right)$) that result from expected prey densities $\Lambda_{c}\left(s\right)$ at the centroid of each foraging habitat and visualize the predicted distribution of prey densities resulting from the Tweedie distribution. This Tweedie distribution will not exactly match the true sampling distribution because it does not exactly account for spatial heterogeneity within the foraging area (i.e., the Tweedie is calculated from prey densities at the centroid of each area, while the true sampling distribution integrates across this area); the magnitude of this mismatch will decrease as habitats become more homogenous within each foraging area. Finally, we also calculate the optimal value for scale (ϕ) and power (p) when assuming that those parameters are constant for all sites and prey species and visualize the predicted distribution of prey densities resulting from that Tweedie GLM approximation. The latter approximation is what can be fitted conveniently using widely available software, so this comparison allows us to visualize the magnitude of errors arising from ignoring fine‐scale spatial heterogeneity and/or specifying a constant value for scale and power parameters.

### Case study demonstrations

We next demonstrate how this approach can be used to solve the inverse problem, that is, provide inference about diet proportions using real‐world data. We use two contrasting case studies:

1. Bill‐load samples from tufted puffins nesting on Middleton Island in the Gulf of Alaska (Hatch & Sanger, 1992). We bin prey into seven major prey taxa, and apply a Generalized Additive Model (GAM) including a log‐linear effect for sea surface temperature 1982–2018 as well as a Gaussian‐process smoother for year for each prey.
2. DNA metabarcoding of prey in scat samples from wolves across an archipelagic landscape in southeast Alaska. We bin prey into eight prey taxa, and fit a model with main effects for prey, a Duchon spline for latitude and longitude (representing spatial variation in area swept) as well as a Duchon spline for latitude and longitude estimated for each prey (Roffler et al., 2021).

See Appendices S4 and S5 for further details regarding data and model structure.

## MODEL FITTING

For all models fitted, we apply GAM fitted using package *mgcv* (Wood, 2006) in the R statistical environment (R Core Team, 2017). We develop a new R package *mvtweedie* that is publicly available (see https://github.com/James‐Thorson‐NOAA/mvtweedie) and that defines an *mvtweedie* S3 class and associated *predict.mvtweedie* function to calculate proportions $\pi_{c}^{*}$ and resulting standard errors given specified values for covariates. We then visualize fits using this predict function and the standard R package *ggplot2* (Wickham, 2016). We provide a simple R script to replicate these analyses as a documented and user‐friendly tutorial that can be adopted for future uses (see Appendix S5 for example code and Data S1 and S2 for demo).

## RESULTS

**FIGURE 1:**
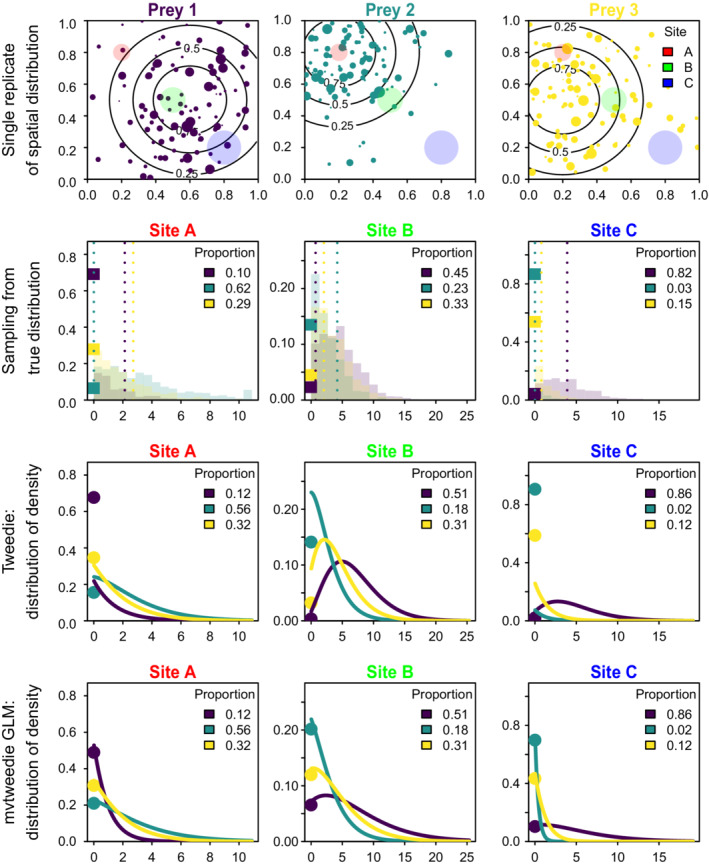
Simulation of prey distribution (top row) for three prey species (labeled Prey 1, Prey 2, and Prey 3), visualizing the prey density field (contour lines, labeled relative to maximum density at the centroid of each prey's range) and location for 1000 individuals of each prey (dots with area proportional to individual mass) with the location of three foraging areas overlayed (labeled Site A, Site B, and Site C). Also showing prey densities from 1000 samples from marked Poisson process (squares showing the proportion of zeros, and a histogram for continuous positive values) of prey biomass from food‐habits sampling (second row) in those three foraging areas (second row), a Tweedie distribution (showing points at zero for the probability of zero, and a line for continuous positive values) calculated when ignoring fine‐scale spatial variation (third row), or a Tweedie distribution when assuming that scale and power parameters are constant for all sites and prey (fourth row). Panels list the true (second row) or predicted (third and fourth rows) proportion from each approximation in the top right of each panel in those rows

The simulated example has highest total prey densities at Site A and decreasing densities for Sites B and C (Figure 1, first row). However, Site A also has smallest foraging area, resulting in approximately similar expected feeding rates for all three sites (*x*‐axis scale of Figure 1, first row). Site A has the highest proportion of Prey 2, while Site B has highest expected proportion for Prey 1, and Site C also has a high proportion for Prey 1. Similarly, Site C has high rates of zero encounters for Prey 2 and 3. Sampling from this prey distribution (Figure 1, second row) shows high rates of encounter for all species in Site B, contrasted with very low encounter rates for Prey 2 and 3 at Site C. A Tweedie distribution ignoring fine‐scale spatial heterogeneity (i.e., evaluated using the true prey density at the center of each foraging area) shows similar distributions to the true sampling distribution, but with small differences in the predicted proportion of each prey (i.e., comparing third and second rows of Figure 1). Finally, the Tweedie GLM approximation (using a single value for ϕ and p) results in some visible differences relative to the Tweedie distribution using sample‐specific parameters, particularly with respect to the predicted proportion of zeros (i.e., comparing height of circles on *y*‐axis of third vs. fourth rows). However, both approaches result in identical predictions for the proportion for each prey. We, therefore, conclude that approximating the marked Poisson process with a Tweedie distribution while ignoring fine‐scale heterogeneity (comparing second and third row) results in a similar magnitude of error as the further approximation using a Tweedie GLM (comparing third and fourth rows).

**FIGURE 2:**
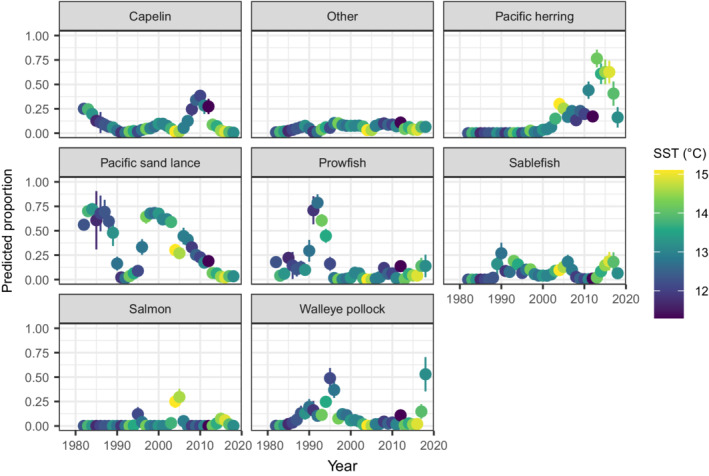
Predicted proportion (*y*‐axis) in each year from 1978 to 2018 (*x*‐axis) for each prey (panels) when fitting bill load samples for Tufted Puffins in Middleton Island, showing both prediction (bullets) and ±SE (whiskers) given the sea surface temperature (SST) in each year (see color legend on right‐hand side)

The resulting model for puffin bill loads shows decadal variability in dominant forage (Figure 2 and Appendix S5: Figure S1). Specifically, Pacific sand lance (*Ammodytes hexapterus*) and prowfish (*Zaprora Silenus*) alternate for highest proportion from 1978–2000, followed by an alternation between Pacific herring (*Clupea pallasii*) and capelin (*Mallotus villosus*) from 2001 to 2018, while salmon (*Oncorhynchus* spp.), pollock (*Gadus chalcogrammus*), and sablefish (*Anoplopoma fimbria*) also show elevated proportions during individual decades. The log‐linear effect of sea surface temperature is significantly negative for prowfish and pollock, and significantly positive for Pacific herring (Appendix S5: Table S3 and Figure S2). The positive effect of sea surface temperature on Pacific herring then explains why herring was a higher proportion of puffin diet in recent warm periods (2001–2007 and 2013–2018), while capelin was a larger component in the intervening cool period (2008–2012).

**FIGURE 3:**
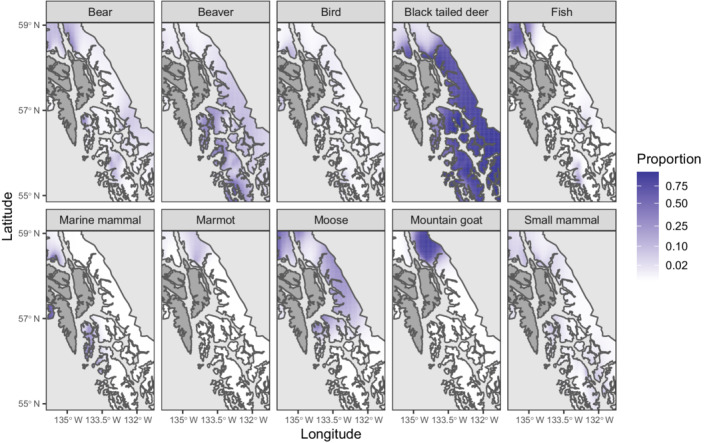
Predicted diet proportion (color scale on right‐hand side) at each longitude–latitude (*x*‐ and *y*‐axes) for each prey (panels) for wolves foraging in southeast Alaska, where predicted proportions are not plotted over water (light gray), Canada (light gray), or islands not inhabited by wolves (Admiralty, Baranof, and Chichagof; dark gray)

As a contrasting example, wolves in southeast Alaska show strong spatial variation in prey proportions in their diet (Figure 3). Specifically, Sitka black‐tailed deer are the dominant prey throughout all areas except the northern edge, where mountain goat and marmot both have higher proportions. Marine mammals show higher proportions along the seaward coastline of northern islands, while moose are a substantial proportion in the central and northern portions and beavers in the central and southern portions. Collectively, these case studies illustrate that spatial and interannual variation can be easily inferred and visualized using this Tweedie GLM while using common statistical and plotting packages.

## DISCUSSION

In this study, we derived a Tweedie distribution for biomass samples from predator food‐habits samples when foraging within a thinned and double‐marked Poisson process. We then showed how to parameterize this distribution using either (1) the thinned biomass density for each prey in Equations ((2), (3), (4)) or (2) the total forage biomass and proportions for each prey in Appendix S3. Finally, we provided an easy‐to‐use R package *mvtweedie* that can implement this approach using GAMs (package *mgcv*) or GLMMs (package *glmmTMB*), while visualizing results using high level methods (package *ggplot2*). We provide two contrasting case studies, which highlight decadal variability in prey species for a central‐place forager (Tufted Puffins) and substantial spatial variability for a generalist carnivore (wolves).

The appropriate interpretation of estimates from the Tweedie GLM will depend upon assumptions about the thinning process occurring during predator foraging. Regardless of thinning processes, the predicted proportions ($\pi_{i}$ from Equation 4) represent diet fractions for those sampled individuals, and analysts could then infer population‐level properties about diet proportions if the sampled individuals are representative of a wider population of predators (Cressie et al., 2009). Alternatively, analysts may seek to interpret variation in diet proportions as indicating variability in the numerical density of those prey species, but this requires additional assumptions about predator thinning rates. Given the thinned point process that underlies this method, prey observed in diets are best understood as a thinned sample from the actual spatial distribution of each prey species. If all prey species of interest are within the diet breadth of an optimally foraging consumer and the most energetically profitable prey are not too abundant, then thinning rates may be constant across space and/or time (Stephens & Krebs, 1987), such that prey proportions would be representative of spatial/temporal variation in density for each.

Analysts increasingly use diets of generalist predators to understand changes in prey availability across time and space when such information may be otherwise limited (Clare et al., 2014; Grüss et al., 2020). Developing exploratory models with covariates (as facilitated by using common packages such as *mgcv*) can improve the accuracy of predictions across space and time, and provide greater understanding of factors regulating trophic interactions including numerical and functional responses. As one example, a Tweedie‐GLM would allow formal tests of prey switching as predators grow. In this case, including an interaction between predator size and prey species (“*formula = ~ … + Size:group*”, involving numeric vector “*Size*” and factor “*group*”) would permit such a test. In this case, a greater estimated slope for one prey than another indicates a progressive increase in proportion for the former prey with predator size, and the resulting response curve could be visualized as a partial dependence or marginal effects plot. Alternatively, a joint model of diet fractions and predator densities supports calculation of predator‐expanded diet fractions. A joint model of diet and predator density therefore can be used to measure landscape‐level consumption, even when analyzing data from monitoring programs wherein diets are sampled in an unbalanced design (Grüss et al., 2020).

Alternatively, the thinning rate in the thinned and marked Poisson process may vary spatially and over time (e.g., if the predator switches from ignoring suboptimal prey in some places or times) and the Tweedie GLM could be adapted to simultaneously estimate spatiotemporal variation in prey densities and thinning rates. However, variation in thinning rate is confounded with variable prey densities; this is analogous to the confounded effect of sampling intensity and population density in presence‐only models (Dorazio, 2014). As in the case of presence‐only models, identifying the effect of a covariate on both thinning rates and prey densities would presumably require integrating data from multiple sampling methods (e.g., diets and prey density surveys). One advantage of the Tweedie GLM is to explicitly acknowledge the confounding between prey densities and thinning rates, while pointing to monitoring and modeling that can separately identify these two processes underlying diet data.

Ecologists increasingly seek to develop integrated models for all available data without needing to transform data prior to analysis (O'Hara & Kotze, 2010). We therefore encourage further use and development of the Tweedie GLM (and the associated multivariate Tweedie distribution, see Appendix S3) as a numerically efficient approximation to the thinned and marked point process that underlies predator foraging. More generally, the Tweedie GLM is appropriate to model multivariate proportions that include 0s and 1s, which arise in many ecological contexts beyond diet samples, and we hope that the *mvtweedie* package facilitates exploration in these cases.

## CONFLICT OF INTEREST

The authors declare no conflict of interest.

## Supporting information


Appendix S1

Appendix S2

Appendix S3

Appendix S4

Appendix S5

Data S1

Data S2

## Data Availability

Data (Thorson et al., 2021a) and code (Thorson et al., 2021b) are available in Dryad (https://doi.org/10.5061/dryad.08kprr53h) and Zenodo (https://doi.org/10.5281/zenodo.5579713), respectively. The R package (Thorson, 2021) documented here is available in Zenodo (https://doi.org/10.5281/zenodo.5579705).
