# Supplementary material for: Diet analysis using generalized linear models derived from foraging processes using R package mvtweedie
Source: Ecology. 2022 Mar 16;103(5):e3637. doi: 10.1002/ecy.3637 (PMC9286827; doi:10.1002/ecy.3637)
Supplement: Supplementary file 1 — Appendix S1 [file ECY-103-0-s003.pdf]

Thorson, Arimitsu, Levi, Roffler. 2022. Diet analysis using generalized linear models derived from foraging processes using R package *mvtweedie*. *Ecology.*

Appendix S1: Details regarding the thinned and double-marked point process model

First, we introduce a Poisson process, where individual prey items are randomly and independently distributed conditional upon intensity $\Lambda(s)$, such that the count of prey encounters, $N(A)$ in area A, from a predator searching in that area follows a Poisson distribution (Illian *et al.*, 2008):

$$N(A) \sim Poisson\left(\int_A \Lambda(s) \mathrm{d}s \right) \qquad \text{Eq. S1}$$

Second, we specifically discuss log-Gaussian Cox processes. These are a convenient special case of point processes, where the log-intensity function varies as a linear function of covariates as well as spatial residuals that follow a Gaussian random field:

$$\log\big(\Lambda(s)\big) = \boldsymbol{\beta}\mathbf{x}(s) + \omega(s) \qquad \text{Eq. S2}$$

where $\omega(s)$ is a Gaussian random field evaluated at location s, $\mathbf{x}(s)$ is the value of covariates at that location, and $\boldsymbol{\beta}$ is the response to those covariates. This log-linked linear predictor allows easy extension involving a combination of fixed and random effects, similar to how generalized linear mixed models are constructed.

Third, we introduce marked point processes, where every individual $k \in \{1,2,\dots,N(A)\}$ that is encountered in a predator having searched area A, $N(A)$, is associated with one or more

additional random variables M_k (called "marks"). Marked point processes typically have two forms:

1. Qualitative ("multi-type") marks, wherein marks arise from a category, $M_{1k} \in \{1,2,\dots,n_c\}$ where n_c is the number of categories (Illian *et al.*, 2004).
2. Quantitative marks, wherein mark M_{2k} has a value drawn from a set of (potentially bounded) real numbers.

For generality in the following, we envision that each prey animal has at least two "marks" which themselves follow some random variable:

A. *Category*: In the following, category (the qualitative mark) represents alternative prey taxa (species, or binned at higher taxonomic levels), where n_c is the number of prey species a predator might encounter and consume (which we call a "species" mark). We envision that the "category" mark will typically be prey taxon (e.g., species), but could be other groupings including size, sex, etc.
B. *Size*: Each prey animal also has a continuous-valued biomass (which we call a "size" mark). The "size" mark might be any characteristic of an individual that is positive and measured on a continuous scale, such as typical biomass within a predator stomach per prey captured, per-capita contribution to chemical marker concentrations within a tissue sample, or DNA reads per local abundance within a sample of environmental DNA.

The interpretation of these marks will clearly vary based on application. For example, food habits samples using stomach-content analysis might be measured in biomass with prey taxa as category, while the size mark might be the relative number of sequence reads when conducting diet analysis with DNA metabarcoding (O'Donovan *et al.*, 2018). This then results in us defining an intensity function $\Lambda_c(s)$ for each prey category, and a separate expectation

$\mathbb{E}(M_{2k}) = W_c(s)$ representing prey animal size, e.g., where the size has expectation $W_c(s)$ for prey type c at location s.

Fourth, we briefly introduce the "thinned" point processes, where some portion of encountered individuals $N(A)$ along with their associated marks are not observed ("censored"). This thinning process arises naturally in many contexts including:

1. Sampling theory, where a proportion of local individuals are not detected by a given sampling method ("detectability");
2. Foraging theory, where a predator may encounter a prey individual within a foraging path but either choose not to capture it or be unsuccessful at capturing it (e.g., due to satiation, handling times resulting from prior captures, likelihood of capture success, or relative preference due to energetic payoff from successful capture).
3. Physiological theory, where a proportion of ingested materials are preferentially metabolized and excreted, or alternatively are integrated into somatic tissue and hence available for later detection by chemical assays.

We collectively refer to the result of these many processes as "thinning", and use $p_c(s)$ to refer to the probability that each individual of prey c and location s is retained (where $1 - p_c(s)$ is the probability that it is censored prior to observation). This thinning process results in food-habits samples that are not representative of prey densities, and future research could develop domain-specific models for these thinning processes to infer changes in prey densities from changes in the composition of food-habits data.

Works cited

Illian, D. J., Penttinen, P. A., Stoyan, D. H., and Stoyan, D. D. 2008. Statistical Analysis and Modelling of Spatial Point Patterns. John Wiley & Sons. 557 pp.

Illian, J., Benson, E., Crawford, J., and Staines, H. 2004. Multivariate methods for spatial point processes–a simulation study. *In* Spatial point process modelling and its applications, pp. 125–130. Ed. by A. Baddeley, P. Gregori, J. Mateu, R. Stoica, and D. Stoyan. University of Jaume.

O'Donovan, S. A., Budge, S. M., Hobson, K. A., Kelly, A. P., and Derocher, A. E. 2018. Intrapopulation variability in wolf diet revealed using a combined stable isotope and fatty acid approach. Ecosphere, 9: e02420.
