# Supplementary material for: Diet analysis using generalized linear models derived from foraging processes using R package mvtweedie
Source: Ecology. 2022 Mar 16;103(5):e3637. doi: 10.1002/ecy.3637 (PMC9286827; doi:10.1002/ecy.3637)
Supplement: Supplementary file 2 — Appendix S2 [file ECY-103-0-s002.pdf]

Thorson, Arimitsu, Levi, Roffler. 2022. Diet analysis using generalized linear models derived from foraging processes using R package *mvtweedie*. *Ecology.*

Appendix S2: The Multinomial-Poisson transformation

In cases where the "size" mark is constant across all individuals for each species, the thinned and marked log-Gaussian Cox model (Appendix S1) reduces to a Poisson distribution with expectation λ_{ic} for every sample i. This allows prey densities to be estimated as follows:

$$N_{ic} \sim Poisson(\lambda_{ic})$$

$$\log(\lambda_{ic}) = \alpha_i + \sum_{k=1}^{n_k} \beta_{kc} x_{ik} \qquad \text{(Eq. S1)}$$

where β_{kc} represent prey-specific responses to covariates x_{ik} associated with each sample i (including an intercept where $x_{i0} = 1$ for all i), and α_i is a normalization parameter for each sample i discussed in detail below.

Importantly, this specification of a log-linked Poisson generalized linear model with multiple categories c provides equivalent estimates to a multinomial logit model for proportions (Birch, 1963; Palmgren, 1981):

$$\mathbf{N}_i \sim Multinomial(\boldsymbol{\pi}) \qquad \text{(Eq. S2)}$$

Where $\boldsymbol{\pi}$ is the vector of proportions π_c and $\mathbf{N}_i$ is the vector of counts N_{ic} for each prey c. The Poisson parameters β_{kc} can be converted to the multinomial logit parameters (and vice-versa) using the formula:

$$\pi_c = \frac{\exp\left(\sum_{k=1}^{n_k} \beta_{kc} x_k\right)}{\sum_{c^*=1}^{n_c} \exp\left(\sum_{k=1}^{n_k} \beta_{kc^*} x_k\right)} \qquad \text{(Eq. S3)}$$

Where this formula transforms log-linear parameters β_{kc} into logit-multinomial parameters π_c given covariates x_k. Estimating parameters for a multinomial logit-model using a log-linked Poisson GLM is sometimes called the "Multinomial-Poisson transformation" (Baker, 1994).

We note that this Multinomial-Poisson transformation involves specifying a coefficient α_i for every sample i within the Poisson distribution (Eq. B1), which we call "normalizing by row" in the following. Treating this coefficient as a fixed effect for every sample is equivalent to conditioning upon the sum $\sum_{c=1}^{n_c} Y_{ic}$, which is done explicitly in a multinomial distribution but done implicitly via estimation of α_i in the Multinomial-Poisson transformation. Alternatively, α_i could:

1. arise from random variation in predator foraging rates and hence be treated as a random effect;
2. arise from known characteristics of each sample i, i.e., treating predator body size as an offset or covariate (Ng *et al.*, 2021);

In essence, α_i is interpreted as an unknown "area" that is foraged prior to sampling, i.e., the product of an unknown size $|A_i|$ of area A_i and thinning rate p_i that is sampled and retained from intensity $\Lambda(s_\mathrm{i})$ from the Poisson process in the vicinity of location s_i. This product $p_i|A_i|$ in turn results in a greater or less value for $\sum_{c=1}^{n_c} Y_{ic}$ for each sample, and results in estimated variability in α_i. In this sense, it is similar to an "area offset" that is included within a log-linked Poisson GLM of $\sum_{c=1}^{n_c} Y_{ic}$ and each constituent element. When estimating $\boldsymbol{\alpha}$ as a fixed effect for each sample i, we impose a corner constraint for identifiability where $\alpha_1 = 0$, such that α_i is the log-ratio of area $p_1|A_i|$ relative to $p_1|A_1|$.

Importantly, including the conditioning upon the total count across categories $\sum_{c=1}^{n_c} Y_{ic}$ (or equivalently estimating α_i as a fixed effect) causes the Poisson GLM to give identical

maximum likelihood estimates of parameters β_{kc} as the multivariate logistic regression (i.e., using a multinomial distribution). In a sense, conditioning upon the total count $\sum_{c=1}^{n_c} Y_{ic}$ causes the estimated proportion $\hat{\pi}_{c_1}$ for category c_1 to have a negative correlation with that of all other categories c_2. This can be seen intuitively, e.g., if a sample has Y_{ic_1} above expectation for category c_1 then expected proportions $\hat{\pi}_c$ will be lower for all other categories.

Re-casting the analysis of composition data as counts arising from this marked Poisson process provides three insights:

1. Fitting a Multinomial model with parameters representing proportions is equivalent (in resulting maximum likelihood and asymptotic covariance estimates) to fitting the Poisson model with parameters representing encounter densities;
2. The parameters for a Poisson distribution have a natural interpretation in terms of densities for individuals that are contributing to the resulting data.
3. The Poisson distribution can easily be augmented to include thinning rates, i.e., the detection probabilities, selection rates, or other processes contributing to the rate at which encountered individuals are assimilated into an aggregate count. If these thinning rates are constant across all samples, then spatial and/or temporal patterns in food-habits samples are representative of patterns in prey densities.

Works cited

Baker, S. G. 1994. The Multinomial-Poisson Transformation. Journal of the Royal Statistical Society: Series D (The Statistician), 43: 495–504.

Birch, M. W. 1963. Maximum Likelihood in Three-Way Contingency Tables. Journal of the Royal Statistical Society. Series B (Methodological), 25: 220–233.

Ng, E. L., Deroba, J. J., Essington, T. E., Grüss, A., Smith, B. E., and Thorson, J. T. 2021. Predator stomach contents can provide accurate indices of prey biomass. ICES Journal of Marine Science. https://doi.org/10.1093/icesjms/fsab026 (Accessed 21 February 2021).

Palmgren, J. 1981. The Fisher information matrix for log linear models arguing conditionally on observed explanatory variable. Biometrika, 68: 563–566.
