# Supplementary material for: Diet analysis using generalized linear models derived from foraging processes using R package mvtweedie
Source: Ecology. 2022 Mar 16;103(5):e3637. doi: 10.1002/ecy.3637 (PMC9286827; doi:10.1002/ecy.3637)

Thorson, Arimitsu, Levi, Roffler. 2022. Diet analysis using generalized linear models derived from foraging processes using R package *mvtweedie*. *Ecology.*

Appendix S3: Interpretation of the multivariate Tweedie distribution

In the main text, we have emphasized the use of a Tweedie GLM to approximate a thinned and marked Poisson process that represents foraging processes. We here discuss an alternative interpretation, involving the definition for a new "multivariate Tweedie" distribution.

We define the multivariate Tweedie distribution as:

$$\mathbf{Y} \sim mvTweedie(\alpha^* \boldsymbol{\pi}, \phi, p) \qquad \text{(Eq. S1)}$$

where **Y** is the vector of responses, $\boldsymbol{\pi}$ is the proportion for each category, and α^* is a variable for each sample. The likelihood for the multivariate Tweedie distribution can be easily calculated from a series of independent Tweedie likelihood calculations (i.e., using Eq. 2-4 in main text). However, defining this as a new multivariate distribution emphasizes several points:

1. As discussed in Appendix B, the Multinomial-Poisson transformation implies that a set of Poisson distributions will yield identical estimates for proportion as a multinomial logit distribution. We have replaced the Poisson distribution with a Tweedie, but there is no name for the ensuing distribution for the vector of Tweedie counts when estimating a proportion (analogous to the Multinomial distribution in the Multinomial-Poisson transformation). We therefore call this new distribution the multivariate Tweedie.
2. We have emphasized in the main text that, when using a multivariate logistic transformation to define proportions $\boldsymbol{\pi}_i$, the variable α_i^* can be calculated as:

$$\alpha_i^* = e^{\alpha_i} \sum_{c=1}^{n_c} \exp\left(\sum_{k=1}^{n_k} \beta_{kc} x_{ik}\right) \quad \text{(Eq. S2)}$$

However, future research could also explore alternative parameterizations for linking covariates x_{ik} to proportions $\boldsymbol{\pi}_i$, and these would presumably not result in a convenient GLM implementation. For example, using the Eq. S1 parameterization and defining $Y_{total} = \sum_{c=1}^{n_c} Y_c$, then $\mathbb{E}(Y_{total}) = \alpha^*$ and an analyst could instead specify a multivariate-probit model for proportions and a log-linked linear model for α^*;

3. The Tweedie GLM developed in the main text fits a single response $\text{vec}(Y_{ic})$ as a function of predictors including prey category c. This implementation does not explicitly require a measurement of Y_{ic} for each category c in each sample i, but this is implicit in our subsequent definition of proportions π_{ic} (in Eq. 4). By contrast, defining the multivariate Tweedie distribution makes this property more explicit;

We therefore see a benefit to describing this joint distribution as a "multivariate Tweedie", and call the associated R package *mvtweedie*. This package approximates standard errors for estimated proportions based on the standard errors for each prey:

$$\widehat{SE}(\pi_{ic}) = \pi_{ic}\left[\frac{\widehat{SE}(\mu_{ic})^2}{{\mu_{ic}}^2} - 2\frac{\widehat{SE}(\mu_{ic})^2}{\mu_{ic}\left(\sum_{c=1}^{n_c}\mu_{ic}\right)} + \frac{\sum_{c=1}^{n_c}\left(\widehat{SE}(\mu_{ic})^2\right)}{\left(\sum_{c=1}^{n_c}\mu_{ic}\right)^2}\right] \quad \text{(Eq. S3)}$$

where this is derived elsewhere (Thorson and Haltuch, 2018). *mvtweedie* is designed to work with R packages *mgcv* (Wood, 2006)*, glmmTMB* (Brooks *et al.*, 2017)*, and VAST* (Thorson and Barnett, 2017; Thorson, 2019), and these packages vary in terms of user familiarity and flexibility of model specification.

Works cited

Brooks, M. E., Kristensen, K., van Benthem, K. J., Magnusson, A., Berg, C. W., Nielsen, A., Skaug, H. J., *et al.* 2017. glmmTMB balances speed and flexibility among packages for zero-inflated generalized linear mixed modeling. The R journal, 9: 378–400.

Thorson, J. T., and Barnett, L. A. K. 2017. Comparing estimates of abundance trends and distribution shifts using single- and multispecies models of fishes and biogenic habitat. ICES Journal of Marine Science, 74: 1311–1321.

Thorson, J. T., and Haltuch, M. A. 2018. Spatiotemporal analysis of compositional data: increased precision and improved workflow using model-based inputs to stock assessment. Canadian Journal of Fisheries and Aquatic Sciences, 76: 401–414.

Thorson, J. T. 2019. Guidance for decisions using the Vector Autoregressive Spatio-Temporal (VAST) package in stock, ecosystem, habitat and climate assessments. Fisheries Research, 210: 143–161.

Wood, S. N. 2006. Generalized additive models: an introduction with R. Chapman and Hall/CRC Press, Boca Raton, FL.

Table S1 – Summary of model flexibility (defined in terms of ability to implement complex extensions to the basic model presented here, listed as rows) for three software packages (columns) for which *mvtweedie* is designed to interface.

	R package		
	mgcv	*glmmTMB*	*VAST*
Include covariates for Tweedie mean μ_{ic}	Yes	Yes	Yes
Include covariates for Tweedie scale ϕ_{ic}	No	Yes	Yes
Include separate value for Tweedie power p_c for each category	No	Yes	Yes

Fig. S1 – Conceptual figure illustrating the relationship between the Poisson, gamma, Tweedie, and multivariate Tweedie distributions outlined in the main text.

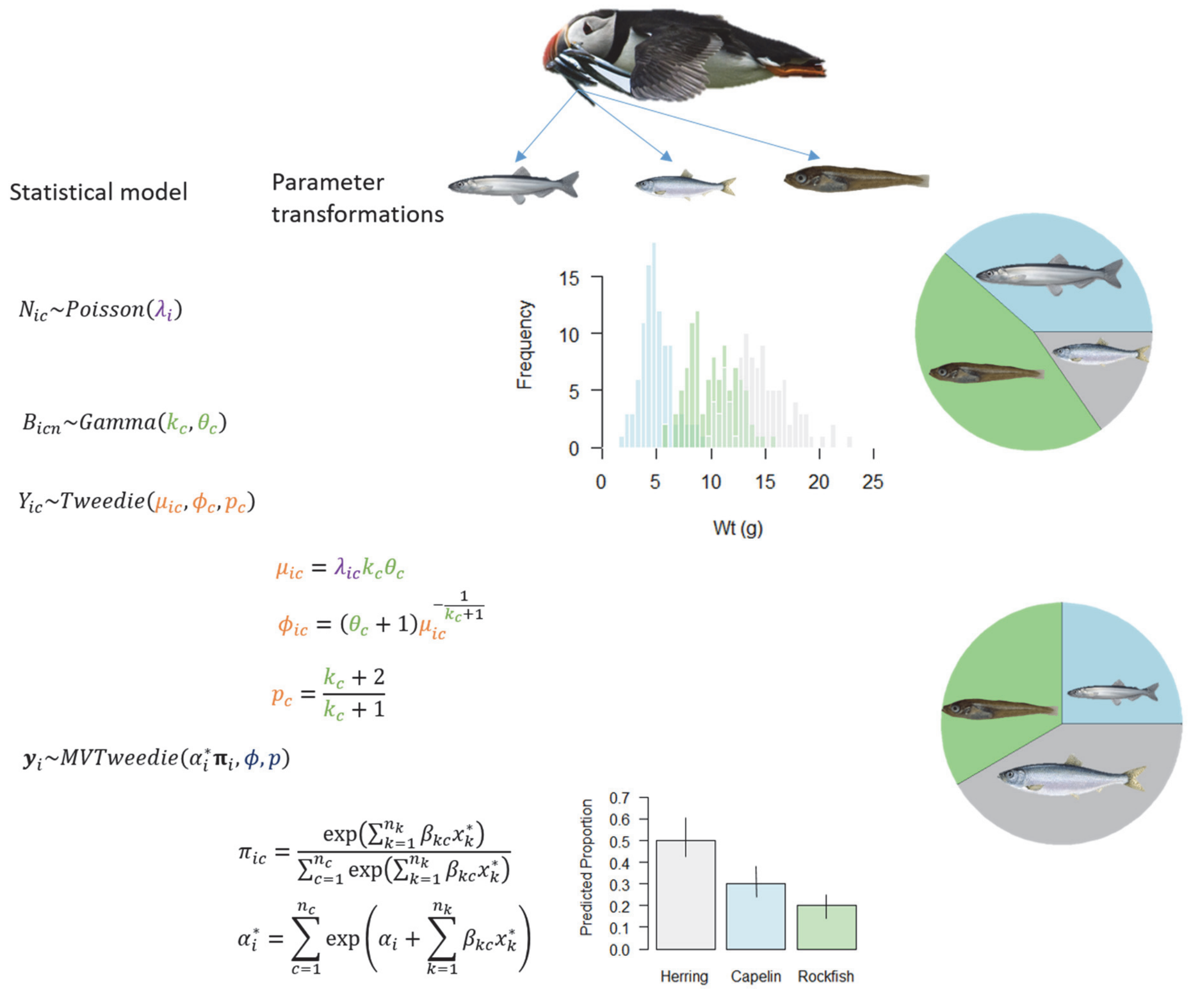

Supplement: Supplementary file 3 — Appendix S3 [file ECY-103-0-s004.pdf]
