# Supplementary material for: Diet analysis using generalized linear models derived from foraging processes using R package mvtweedie
Source: Ecology. 2022 Mar 16;103(5):e3637. doi: 10.1002/ecy.3637 (PMC9286827; doi:10.1002/ecy.3637)
Supplement: Supplementary file 4 — Appendix S4 [file ECY-103-0-s005.pdf]

Thorson, Arimitsu, Levi, Roffler. 2022. Diet analysis using generalized linear models derived from foraging processes using R package *mvtweedie*. *Ecology.*

Appendix S4: Details regarding case studies

We specifically use two contrasting case studies:

1. Bill-load samples from tufted puffins nesting in Middleton Island in the Gulf of Alaska. We bin prey into seven major prey taxa, and apply a Generalized Additive Model including a log-linear effect sea surface temperature 1982-2018 (Huang *et al.*, 2020) as well as a Gaussian-process smoother for year for each prey. Tufted puffins are known to forage 50-100 km from their breeding colonies and can dive to depths of 135 m to capture prey (Piatt *et al.*, 2018). They deliver whole, fresh fish back to provision chicks in their burrows. Samples are collected by placing a screen to block the burrow entrance, which causes the adult to drop the meal. We characterized puffin diet composition and mass by taxa with 572 samples across 25 years between 1978-2018 (Hatch and Sanger, 1992). Samples included one to five different prey taxa (mean = 2, sd = 1), and ranged from 1 to 165 prey items per sample (mean = 10, SD = 17). We then focus inference on interannual variation in prey composition, while also including a linear effect of surface temperature records in the oceans surrounding Middleton Island.
2. DNA metabarcoding of prey in scat samples from wolves across an archipelagic landscape in southeast Alaska. We bin prey into eight prey taxa, and fit a model with main effects for prey and year as well as a random-effect for their interaction. Wolves are generalist apex terrestrial predators and are widely recognized for their dietary plasticity. We characterized dietary diversity of wolves by collecting 860 scats across the Southeast Alaskan wolf range

during 2010–2018 and identifying vertebrate prey species using metabarcoding of amplified target DNA sequences (Roffler *et al.*, 2021). We quantified wolf diet composition using relative read abundance (RRA) calculated as the mean proportion of DNA sequence reads from each species among scat samples. RRA proportions of dominant prey species are correlated with the volume of diet item per scat estimated from mechanical sorting (Massey *et al.*, 2021). The scat samples contained 55 diet items representing species from 42 genera and 29 families which we collapsed into 8 prey. Scat samples on average contained 47,215 diet item DNA sequences per sample (SE = 2353), and one to eight diet items per scat (mean = 1.40, SD = 0.741). We then focus analysis on spatial variation in prey consumption.

Works cited

Hatch, S., and Sanger, G. 1992. Puffins as samplers of juvenile pollock and other forage fish in the Gulf of Alaska. Marine Ecology Progress Series, 80: 1–14.

Huang, B., Liu, C., Banzon, V. F., Thorne, P. W., Freeman, E., Graham, G., Hankins, B., *et al.* 2020. NOAA 0.25-degree Daily Optimum Interpolation Sea Surface Temperature (OISST). NOAA National Centers for Environmental Information. https://doi.org/10.25921/RE9P-PT57.

Massey, A., Roffler, G., Vermeul, T., Allen, J., and Levi, T. 2021. Comparison of mechanical sorting and DNA metabarcoding for diet analysis with degraded wolf scats. Ecography: 2019.12.13.875898.

Piatt, J. F., Arimitsu, M. L., Sydeman, W. J., Thompson, S. A., Renner, H., Zador, S., Douglas, D., *et al.* 2018. Biogeography of pelagic food webs in the North Pacific. Fisheries Oceanography, 27: 366–380.

Roffler, G. H., Allen, J. M., Massey, A., and Levi, T. 2021. Wolf Dietary Diversity in an Island Archipelago. Bulletin of the Ecological Society of America, 102: 1–6.
