# Supplementary material for: Diet analysis using generalized linear models derived from foraging processes using R package mvtweedie
Source: Ecology. 2022 Mar 16;103(5):e3637. doi: 10.1002/ecy.3637 (PMC9286827; doi:10.1002/ecy.3637)
Supplement: Supplementary file 5 — Appendix S5 [file ECY-103-0-s007.pdf]

Thorson, Arimitsu, Levi, Roffler. 2022. Diet analysis using generalized linear models derived from foraging processes using R package *mvtweedie*. *Ecology.*

Appendix S5: Additional figures/tables for case-studies

Fig. S1: Same as Fig. 2 in main text, but also showing the number of bill-load samples with a given proportion of each prey in each year (grey circles, with size shown on right-hand-side)

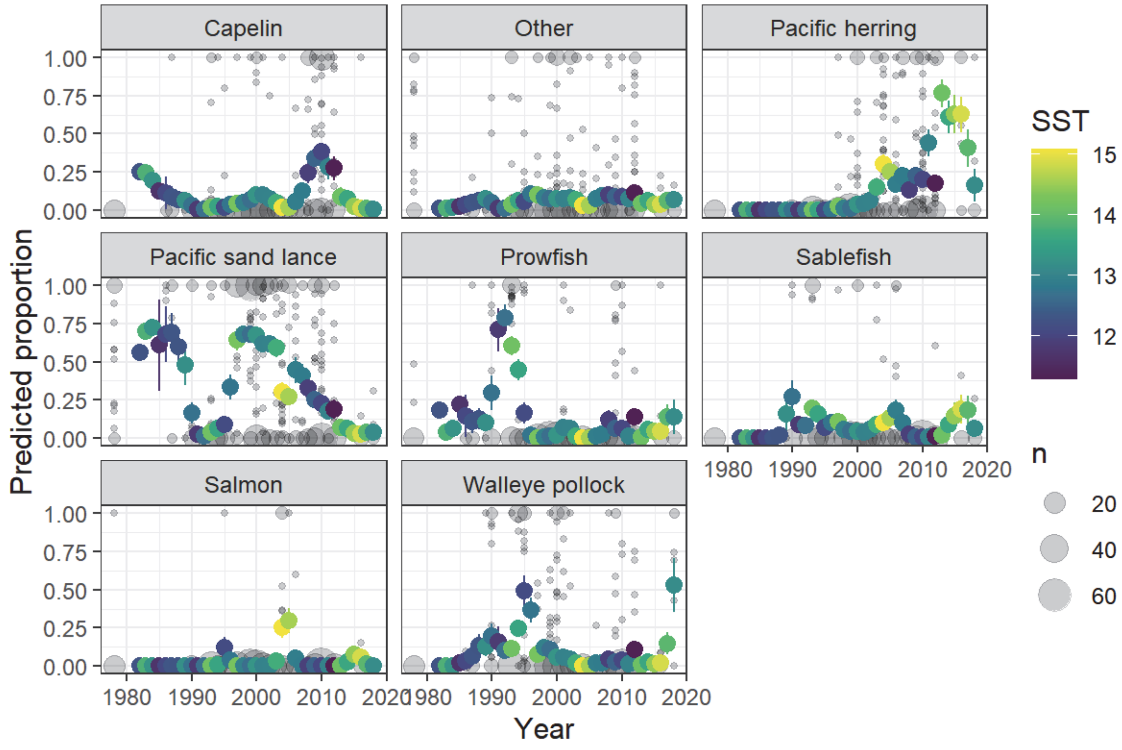

Fig. S2: Graphical representation of estimated log-linear effect of sea surface temperature (SST) on consumption for each prey species (showing maximum likelihood estimate +/- twice the standard error); see Table S3 for details and exact values.

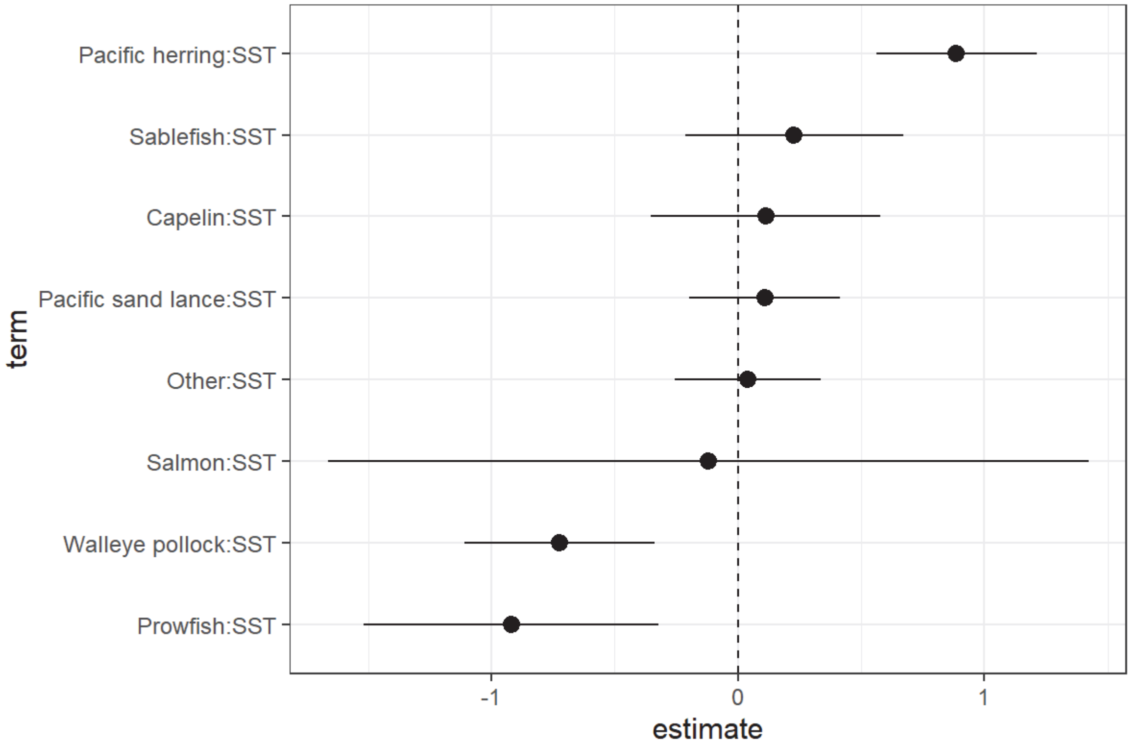

Table S1: Structure for two case studies when fitted using the Tweedie generalized additive model using *mgcv* (see Table S2 for example syntax). In this code, Response a numeric vector of responses for each sample i, group is a factor representing prey species for each sample, Year, Latitude, and Longitude are numeric vectors indicating Year and location for each sample, SST is a numeric vector indicating sea surface temperature associated with each sample, SST:group indicates that the model should estimate an interaction between SST and group such that each prey species has a different log-linear response to SST, s(Year,by=group,bs="gp") indicates that the model should estimate a Gaussian process smoother for Year separately for each prey species, s(Latitude,Longitude,m=c(0.5,1),bs=”ds”) indicates that the model should estimate a Duchon-spline smoother for spatial variation in area-swept, and s(Latitude,Longitude,by=group, m=c(0.5,1),bs=”ds”) indicates the model should estimate spatial variation in prey consumption for each prey species separately.

Case study	Formula for mean (μ_{ic})
Tufted puffin in Middleton Island	formula = Response ~ 0 + group + s(Year,by=group,bs="gp") + SST:group
Wolves in Southeast Alaska	formula = Response ~ 0 + group + s(Latitude,Longitude,m=c(0.5,1),bs=”ds”) + s(Latitude,Longitude,by=group, m=c(0.5,1),bs=”ds”)

Table S2: Demonstration of code required for future users:

Purpose	R code
Load and format data	data(Middleton_Island_TUPU, package="mvtweedie")
Run model	fit = gam(formula = Response ~ 0 + group, data = Middleton_Island_TUPU, family = tw)<br>class(fit) = c("mvtweedie", class(fit))
Predict response	out = predict(fit, origdata = DF)<br>out$lower = out$fit - out$se.fit<br>out$upper = out$fit + out$se.fit
Plot output	ggplot(out, aes(group, fit)) +<br>geom_pointrange(aes(ymin = lower, ymax = upper))

Table S3 – Coefficient estimates fitting a Generalized Additive Model to bill load samples of food habits for eight prey of tufted puffins in Middleton Island, including an intercept and slope representing the log-linear effect of sea-surface temperature (in degrees Celcius) on thinned densities for each prey; we show the estimate and standard error (columns 3-4), as well as the t-value and resulting *p*-value using a two-sided Wald test (showing slopes that are significant at a 0.05 level in bold)

Parameter	Prey	Estimate	SE	t value	*p*-value
Slope for log-linear effect of Sea Surface Temperature	Pacific sand lance	0.108	0.153	0.708	0.479
	Prowfish	**-0.920**	**0.299**	**-3.080**	**0.002**
	Other	0.040	0.149	0.268	0.789
	Salmon	-0.121	0.772	-0.157	0.875
	Walleye pollock	**-0.724**	**0.193**	**-3.742**	**<0.001**
	Capelin	0.112	0.233	0.482	0.630
	Sablefish	0.228	0.221	1.031	0.302
	Pacific herring	**0.887**	**0.163**	**5.458**	**<0.001**
Intercept	Pacific sand lance	-0.290	2.004	-0.145	0.885
	Prowfish	10.935	3.812	2.868	0.004
	Other	-0.812	1.951	-0.416	0.677
	Salmon	-10.457	10.844	-0.964	0.335
	Walleye pollock	8.878	2.497	3.556	<0.001
	Capelin	-1.679	3.042	-0.552	0.581
	Sablefish	-3.876	2.902	-1.335	0.182
	Pacific herring	-12.819	2.179	-5.882	<0.001
