# Supplementary material for: Diet analysis using generalized linear models derived from foraging processes using R package mvtweedie
Source: Ecology. 2022 Mar 16;103(5):e3637. doi: 10.1002/ecy.3637 (PMC9286827; doi:10.1002/ecy.3637)
Supplement: Supplementary file 6 — Data S1 [file ECY-103-0-s001.zip › MetadataS1.pdf]

Thorson, Arimitsu, Levi, Roffler. 2022. Diet analysis using generalized linear models derived from foraging processes using R package *mvtweedie*. *Ecology.*

Data S1

Code to reproduce analysis and figures.

Authors of the material provided in DataS1.zip

James T. Thorson
AFSC, NMFS, NOAA
7600 Sand Point Way NE, Seattle, WA 98115, USA
James.Thorson@noaa.gov

Mayumi L. Arimitsu
U.S. Geological Survey Alaska Science Center
250 Egan Dr., Juneau, AK 99801, USA
marimitsu@usgs.gov

Taal Levi
Department of Forest Ecosystems and Society
OSU, Corvallis, OR 97331, USA
Taal.Levi@oregonstate.edu

Gretchen H. Roffler
Alaska Department of Fish and Game
802 3rd Street, Douglas, AK, 99824, USA
gretchen.roffler@alaska.gov

File list (files found within DataS1.zip)

```
Reproducible_script_R1.R
Wolf.csv
Seabird.csv
MDO.seabirdforagingarea.SST.csv
```

Description

`Reproducible_script_R1.R` – R script used to replicate all analysis and figures in main text and appendices. See comments at top for directions prior to running.

`Wolf.csv` – CSV file containing four columns used in the wolf metabarcoding case-study in Fig. 3 of the main text:

1. "Latitude" -- Latitude of scat sample in Degree-decimals;
2. "Longitude" -- Longitude of scat sample;
3. "group" -- prey taxonomic group used in analysis;
4. "Response" -- metabarcoding read count used as response variable.

`Seabird.csv` – CSV file containing three columns used in the seabird bill-load case-study in Fig. 2 of the main text:

1. "Year" – Year AD for bill-load sample;
2. "group" -- prey taxonomic group used in analysis;
3. "Response" – bill-load count used as response variable.

`MDO.seabirdforagingarea.SST.csv` – CSV file containing two additional columns used in the seabird bill-load case-study in Fig. 2 of the main text:

1. "Year" – Year AD, including all Years used in Fig. 2;
2. "SST_mean" – average sea surface temperature near Middleton Island;
