# Supplementary material for: Diet analysis using generalized linear models derived from foraging processes using R package mvtweedie
Source: Ecology. 2022 Mar 16;103(5):e3637. doi: 10.1002/ecy.3637 (PMC9286827; doi:10.1002/ecy.3637)
Supplement: Supplementary file 7 — Data S2 [file ECY-103-0-s006.zip › MetadataS2.pdf]

Thorson, Arimitsu, Levi, Roffler. 2022. Diet analysis using generalized linear models derived from foraging processes using R package *mvtweedie*. *Ecology.*

Data S2

Static copy of R package used for analysis, with code to install included in R script in Data S1.

Author of the material provided in DataS2.zip

James T. Thorson
AFSC, NMFS, NOAA
7600 Sand Point Way NE, Seattle, WA 98115, USA
James.Thorson@noaa.gov

File list (files found within DataS2.zip)

```
data
man
R
DESCRIPTION
LICENCE
NAMESPACE
README.md
```

Description

`data` – Directory containing data installed with R package
`man` – Directory containing documentation for functions
`R` – Directory containing functions
`DESCRIPTION` – Description of R package
`LICENCE` – License for R package
`NAMESPACE` – List of functions in format expected in R package
`README.md` – Readme file used in GitHub to create landings page.
